# Subclinical Upper Eyelid Ptosis in Asian Patients: The Role of Levator Advancement in Optimizing Outcomes in “Cosmetic” Upper Blepharoplasty

**DOI:** 10.1007/s00266-023-03697-1

**Published:** 2023-10-11

**Authors:** Chin-Ho Wong, Michael Ku Hung Hsieh, Bryan Mendelson

**Affiliations:** 1 W Aesthetic Plastic Surgery, #06-28/29, Mount Elizabeth Novena Specialist Center, 38 Irrawaddy Road, Singapore, 329563 Singapore; 2 Department of Plastic Reconstructive & Aesthetic Surgery, KK Women’s and Children’s Hospital, Singapore, Singapore; 3 Department of Plastic Reconstructive and Aesthetic Surgery, Singapore General Hospital, Singapore, Singapore; 4 The Centre for Facial Plastic Surgery, Toorak, Victoria Australia

**Keywords:** Upper blepharoplasty, Functional, Cosmetic, Oriental, Lids, Eyelids, Forehead wrinkles

## Abstract

**Background:**

*Subclinical ptosis* is prevalent in Asian patients presenting for aesthetic upper blepharoplasty. To achieve predictable and satisfactory results in these patients, addressing the ptosis component is critical. In this paper, we present a precision levator advancement technique that enabled us to predictably incorporate the levator advancement into our upper blepharoplasty to deliver more predictable results in these patients.

**Materials and Methods:**

Asian patients with normal or near normal margin to reflex distance 1 (MRD 1 of ≥ 3.5 mm) and symptoms and signs of straining of the frontalis with eyelid opening were diagnosed with subclinical upper eyelid ptosis and included in this prospective study. The advancement required was estimated pre-operatively using a formula that we developed. Our surgical technique is presented in detail here, and our long-term results were analysed.

**Results:**

From December 2019 to August 2022, 97 patients were included in this study. Sixty-five patients were primary cases and 32 were revision cases. The mean follow-up was 15 months. Of the 192 eyelids analysed, our formula was able to correctly identify the required fixation location in 69% of eyelids. In majority of the eyelids (94%), the correct location of fixation location within +/− 1 mm of the estimated location. All patients (100%) were satisfied with their long-term results. Our revision rate was 3%.

**Conclusions:**

Incorporating a precisely done levator advancement into the upper blepharoplasty in patients with subclinical ptosis is critical for optimizing the aesthetic *and* functional outcomes. This approach has enabled us to perform this procedure greater predictably in this group of patients.

**Level of Evidence III:**

This journal requires that authors assign a level of evidence to each article. For a full description of these Evidence-Based Medicine ratings, please refer to the Table of Contents or the online Instructions to Authors www.springer.com/00266.

**Supplementary Information:**

The online version contains supplementary material available at 10.1007/s00266-023-03697-1.

## Introduction

“Asian upper blepharoplasty” is one of the most common cosmetic procedures done in Asia [1, 2]. The aim of the surgery is to create attractive and brighter eyes. Much of the emphasis in the literature on Asian upper blepharoplasty focuses on fold creation techniques, to achieve the desired height, symmetry and shape of the upper eyelid crease [1–4]. Many papers noted that creating the double eyelid crease in patients without one gives the visual *illusion* of widening of the palpebral aperture [5–7]. However, the actual widening or *optimization* of the palpebral apertures to deliver better aesthetic *and* functional results are rarely discussed in cosmetic Asian upper blepharoplasty. In *Caucasian patients*, blepharoplasty with concurrent levator palpebrae superioris (LPS)/Muller muscle surgery to widen the palpebral aperture to deliver better aesthetic *and* functional results is an accepted concept. Carraway was a proponent for blepharoplasty with concurrent upper eyelid ptosis correction as early as the late 1980s [8, 9]. This concept was also advocated by many other surgeons [10–12]. Massry refers to a “*cosmetic* ptosis repair” and Martin similarly discussed the foundation of good upper lid surgical results as the requirement for “ptosis repair in *aesthetic* blepharoplasty” [11, 13].

Because Asian upper blepharoplasty is quite different from Caucasian upper blepharoplasty, this concept of optimizing function with upper blepharoplasty has not been widely emphasized or practised in the former [14]. However, leading thinkers in Asian upper blepharoplasty have increasingly stressed the need to optimize function as a prerequisite for satisfactory results. Chen noted that in Asian upper blepharoplasty, “decreased levator function and latent ptosis are commonly missed”, with the recommendation that “even mild 1–2 mm ptosis should be separately corrected before any attempt to create a double eyelid crease” for ideal results [15]. This is necessary because having the qualities of attractive or beautiful eyes are all built upon *optimally functioning levator mechanisms* of the upper eyelids [1, 4, 6, 16]. With this critical prerequisite of the attractive upper eyelid fulfilled, other cosmetic aspect of the upper eyelid such as the height, crispness and symmetry of the eyelid crease may be more reliably and predictably achieved.

While the benefits of incorporating the levator advancement into the upper blepharoplasty of Asian patients with subclinical ptosis is increasingly accepted, to achieve the desired results, the surgeon would have to *upgrade* their procedure from a “simple” cosmetic upper blepharoplasty to a *cosmetic and functional upper blepharoplasty*. There are risks inherent to this procedure, specifically causing asymmetry to the palpebral aperture from over- or under-correction. The clinical threshold for more widespread adoption of this approach would be the development of technical advancements that deliver greater precision while minimizing complications. We have previously published our approach to the levator advancement procedure which allows us to perform this procedure with greater predictability and low revision rates [17–20]. This approach may be applied to patients with subclinical ptosis, both in primary or revisional cases. This paper details our experience and outcomes with this approach in this cohort of patients.

## Patient Selection

Patients who presented for upper blepharoplasty and met the following inclusion criteria were diagnosed with subclinical ptosis and included in this study:

1. Asian ethnicity
2. Normal or near normal palpebral aperture: Margin to reflex distance 1 (MRD 1) of + 4.5 mm (normal aperture) to + 3.0 mm (mild ptosis of < 1.0 mm) [21, 22].
3. Excellent or good levator function (≥ 10 mm).
4. Symptoms of heaviness, straining or difficulty with opening their upper eyelids
5. Signs of unilateral or bilateral frontalis activation with eye opening: Frontalis straining was quantitated by brow elevation with eyelid opening [17].

Patient with more significant degree of ptosis (MRD 1 < 3 mm) were excluded. Patients with pre-existing lid retraction were also excluded from this study. The cohort of patients included in this study would be one that would conventionally be offered cosmetic upper blepharoplasty only *without* any levator manipulation. Only patients with at least 1-year follow-up were included in this study.

## Material and Methods

From December 2019 to August 2022, 97 patients were prospectively included in this study. In total, 192 upper eyelids (2 patients had unilateral procedure) were analysed in this study. Sixty-five patients were primary cases and 32 were revision cases. There were 79 female patients and 18 male patients. The mean age of the patients were 41 years of age (range 16–68). The mean follow-up time was 15 months (range 12–35). Standard pre- and post-operative photographs were used for comparison. Patients subjectively rated the outcomes of their surgery with a Likert-type scale of eyelid appearance: 0—worse; 1—unchanged; 2—improved; and 3—markedly improved.

### Pre-Operative Determination of Levator Advancement Fixation Location

We have previous published our method of pre-operatively estimating the location of suture placement relative to the musculoaponeurotic junction (MAJ) of the levator of the upper eyelid [17, 18]. Table 1 shows our *updated* formula. To increase the precision of our estimation, we have refined our elevation of the upper eyelid required (A) into incremental categories of + 0.5 mm. Brow elevation (B) has been refined into finer subcategories to reflect a greater range of clinical manifestations of the degree of frontalis strain (please see Video #1 that explains our assessment of brow elevation) [23]. This system of assessment was used to evaluate out patients pre-operatively and the estimated locations of suture placement determined. Please see Video #2 that illustrates the pre-operative planning for our surgical demonstration patient.

**Table 1:** Our formula for determining the levator advancement needed for Asian patients

Ptosis correction needed (A)	Brow elevation with eye opening (B)	Eye dominance (C)
0 mm: − 5.0	Absent: + 0	Dominant eye: + 0
0.5 mm: − 4.5	Mild minus: + 0.5	Co-dominant eye: + 0
1 mm: − 4.0	Mild: + 1	Non-dominant EYE: + 1
1.5 mm: − 3.5	Moderate minus: + 1.5	
2 mm: − 3.0	Moderate: + 2	
2.5 mm: − 2.5	Severe minus: + 2.5	
3 mm: − 2.0	Severe: + 3	
3.5 mm: − 1.5		
4 mm: − 1.0		
4.5 mm: − 0.5		
5 mm: 0		

These values are referred from the musculoaponeurotic junction (MAJ) of the levator of the upper eyelid, with a value of 0 mm denoting the location of the MAJ and negative and positive values denoting distance below and above this landmark, respectively. Parameter A is the amount of upper eyelid elevation needed, B is the degree of brow elevation present with eye opening and C is for eye dominance. Summation of these 3 parameters gives a value that is the expected levator advancement needed for that eyelid

Estimated distance (in mm) from the musculoaponeurotic junction to anterior tarsus for the levator advancement = A + B + C

### Surgical Technique

Markings: The lower incision is marked at 8.0–9.5 mm from the ciliary margins with the skin under slight tension. Skin excision is done very conservatively and kept within the zone of thin skin in the upper eyelids and ranges from 2 to 8 mm. The locations of the mid-pupil line, medial and lateral corneal–scleral limbus are marked. These are the horizontal fixation locations for our levator advancement sutures. The procedure may be done under local anaesthesia or light intravenous sedation. Local anaesthetic consisted of a mixture of 10 cc 1% lignocaine, 10 cc 1% Ropivacaine and 0.1 cc of 1:1000 adrenaline (giving a dilution of 1:200,000 of the concentration of the adrenaline in the anaesthetic mixture). Approximately 1.0 cc of the local anaesthesia is administered per upper eyelid.

**Fig. 1:**
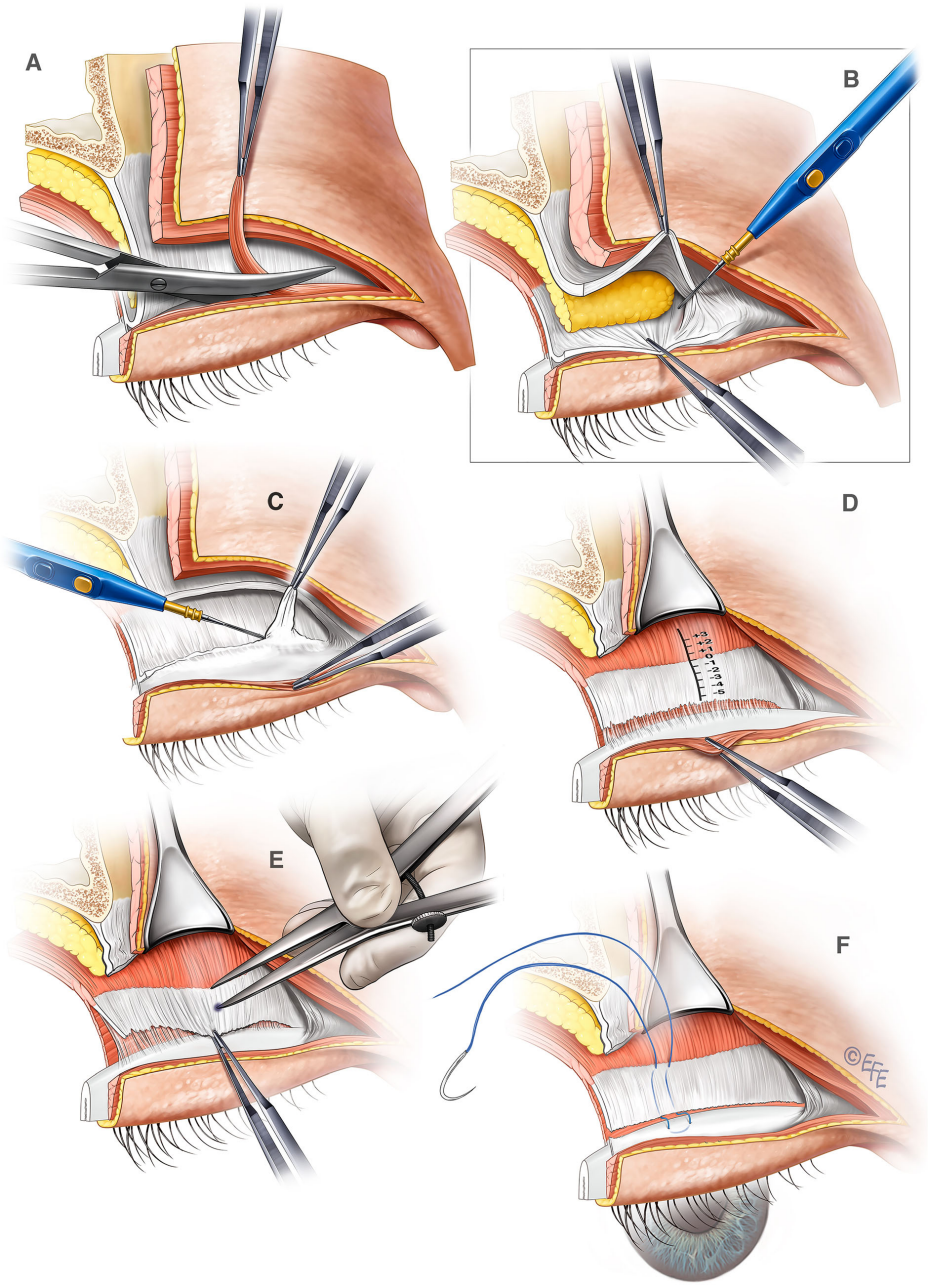
Key steps of our procedure. **A** A trapezoid strip of pre-tarsal orbicularis oculi is precisely excised. This creates a precise gap to allow the dermis to adhere to the lower edge of the levator aponeurosis upon closure to create a crisp upper eyelid crease. **B** With the orbital septum under tension, the orbital septum is carefully cut across the upper eyelid to expose the levator mechanism. **C** The fibrofatty tissue at the upper edge of the tarsus is carefully excised to clearly visualize the upper edge of the tarsus. **D** The musculoaponeurotic junction (MAJ) is the key landmark for our levator advancement. The location of the MAJ is designated location 0 mm, and positive and negative values of advancement would be above and below the MAJ, respectively. **E** As determined by our formula, the location of suture placement is precisely marked with a fine calliper with its tips dipped in methylene blue. **F** The first suture is placed at the mid-pupil line with a round body 6/0 Prolene to achieve the levator advancement

Video #3 demonstrates our surgical technique [17]. After skin excision, the pre-tarsal orbicularis oculi is lifted off the levator mechanism. A strip of pre-tarsal orbicularis is excised (Fig. 1a). The orbital septum is then carefully opened. The landmark to identify here is the discrete fat pad that is the lateral extension of the central upper eyelid fat pad. Below this, the lower edges of the levator aponeurosis will be visible as a distinctive “*white line*” [24]. Once identified, the assistant then picks up and retracts the lower edge of the levator aponeurosis caudally. With the surgeon picking up the orbital septum to provide the countertraction to keep the orbital septum under slight tension, the orbital septum may then be safely and completely open across the upper eyelid to expose the levator aponeurosis and the *musculoaponeurotic junction* (Fig. 1b). The fibrous tissue that is the fusion of the orbital septum and the levator aponeurosis may be conservatively excised over the expected fixation points for the levator advancement to enable clear visualization of the upper edge of the tarsus (Fig. 1c).

According to the pre-operatively determined advancement location, the advancement point is precisely marked on the levator relative to the musculoaponeurotic junction (Fig. 1d and e). A 6/0 double arm non-cutting suture was used for this purpose (Ethicon Inc. reference 8610H). The suture is placed from this point through the levator aponeurosis or muscle (staying above the Muller muscle). A firm bite is then taken of the tarsus 2 mm below the upper edge directly below the entry point on the levator and then passed back to the same location on the levator approximately 2 mm medial to the entry point (Fig. 1f). The suture is then firmly tied. The procedure is then repeated on the contralateral eyelid and a levator advancement fixation suture placed in a similar manner. The patient is then sat up and asked the open the eyelids. The adequacy of the palpebral aperture and symmetry is checked. The palpebral aperture should be exactly the intended correction without over- or under-correction. If the aperture is suboptimal, the levator advancement location may be adjusted as needed. If the palpebral aperture is satisfactory, the levator advancement may then be reinforced at the level of the medial and lateral corneal–scleral limbus at the same distance from the musculoaponeurotic junction (MAJ) with 2 more fixation sutures. In total, three levator advancement fixation sutures are placed per upper eyelid. After placement of all three sutures, the patient is then sat up for a final check for adequacy and symmetry of the palpebral aperture before final closure. The dermis is then sutured to the lower edge of the levator aponeurosis with a 7/0 Vicryl suture to create a crisp upper eyelid crease. This was reinforced with the skin closure with 6/0 Ethilon suture in a skin–levator–skin manner.

## Results

**Fig. 2:**
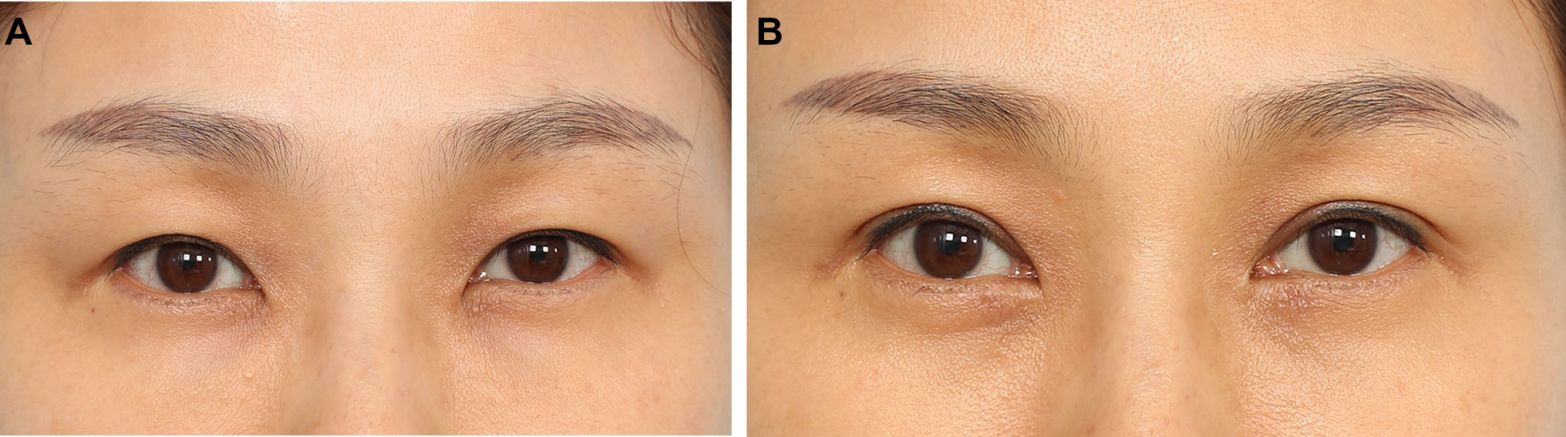
**A** and **B** A 42-year-old female presented for upper blepharoplasty. On examination, her MRD 1 was + 4.5 mm bilaterally. Her brow was stable on the right. On her left, she has moderate minus brow elevation with eyelid opening. She was right eye dominant. Her pre-operative estimated fixation locations were − 5.0 mm (− 5.0 + 0 + 0) and − 2.5 mm (− 5.0 + 1.5 + 1.0) on the right and left, respectively. Intraoperatively fixation was done at – 5 mm and − 2.5 mm on the right and left, respectively. A medial epicanthoplasty was performed at the same time. Here she is shown at 1 year post-op with good symmetry and height of the palpebral aperture, symmetric upper eyelid crease and elimination of the slight frontalis strain that was present pre-operatively. Patient reported that she was able to open her eyes more effectively after the procedure

**Fig. 3:**
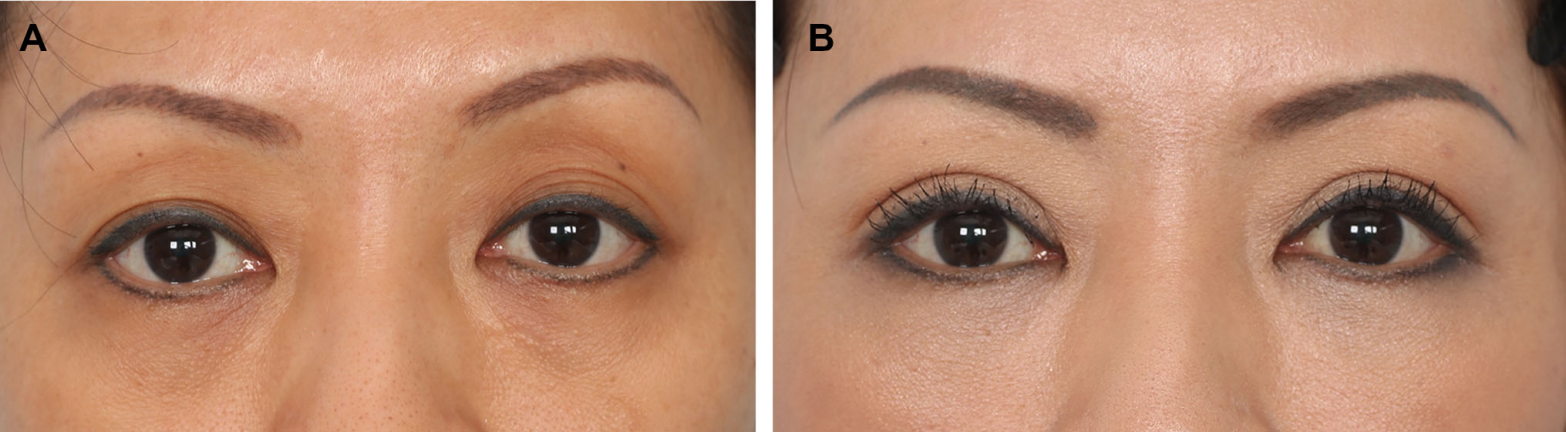
**A** and **B** A 46-year-old female present difficulty opening her upper eyelids. On examination, her MRD 1 was + 4.0 mm bilaterally. Her brow elevation was mild on the right and moderate on the left. She was right eye dominant. Her pre-operative estimated fixation was − 3.5 mm (− 4.5 + 1.0 + 0) and − 1.5 mm (− 4.5 + 2.0 + 1.0) on the right and left, respectively. Intraoperatively, adequate and symmetrical aperture was achieved at − 3.5 mm and − 1.5 mm on the right and left upper eyelid, respectively. She also underwent extended transconjunctival lower blepharoplasty at the same time. Here she is shown at 1 year post-surgery with adequate and symmetrical upper eyelid crease. Her eye brows have relaxed to a more aesthetic location and the upper eyelid hollowing resolved. She reported that her symptoms of straining and difficulty with opening her upper eyelids resolved after the procedure

**Fig. 4:**
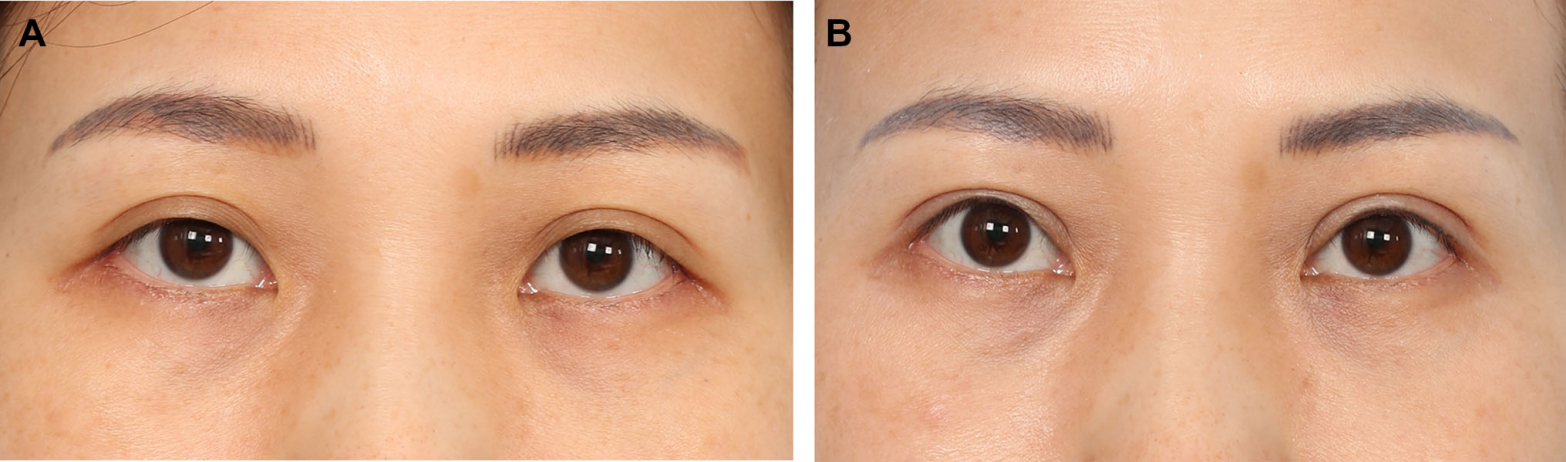
**A** and **B** A 39-year-old female presenting for upper blepharoplasty. Her MRD 1 was + 3.5 mm bilaterally and she has mild and mild minus brow elevation with eye opening on the right and left upper eyelid, respectively. She was right eye dominant. Her estimated fixation locations was − 3.0 mm (− 4.0 + 1.0 + 0) and − 2.5 mm (− 4.0 + 0.5 + 1.0) on the right and left, respectively. Intraoperatively, good aperture and symmetry of the upper eyelid were achieved at − 2.0 mm and − 2.0 mm on the right and left upper eyelid, respectively. Here she is shown at 1 year post-surgery. Her aperture is optimized, her upper eyelid crease is crisp and symmetrical, and her brows have relaxed to a more aesthetic location

**Fig. 5:**
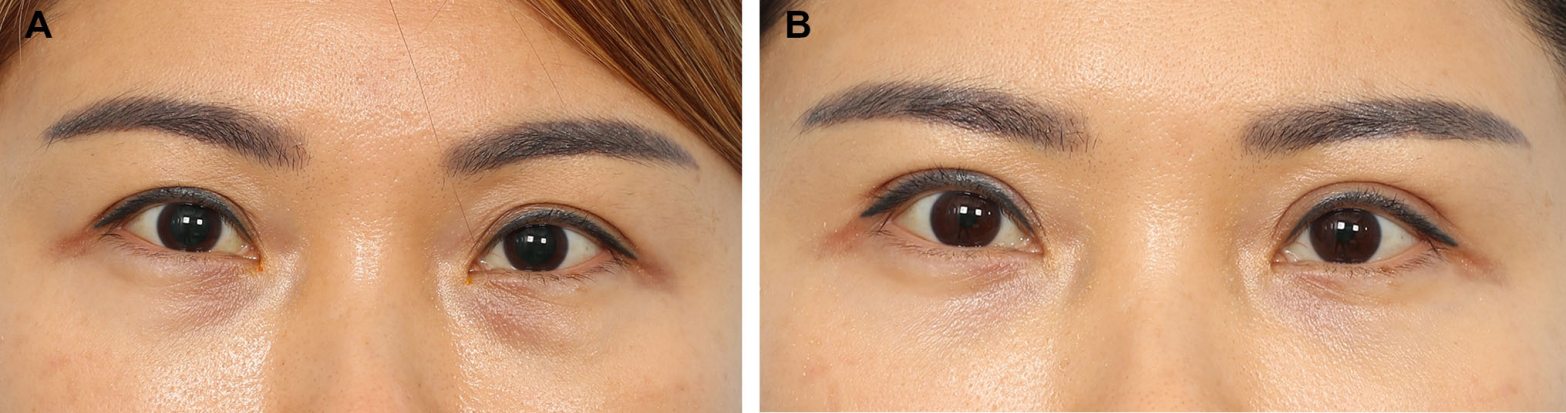
**A** and **B** This 47-year-old female underwent upper blepharoplasty and extended transconjunctival eye bag removal. Her pre-operative assessment and surgery is shown in Videos #2 and #3, respectively. Her recovery and long-term functional outcomes are shown in Video #4. Her 1-year post-surgery result is shown here

**Fig. 6:**
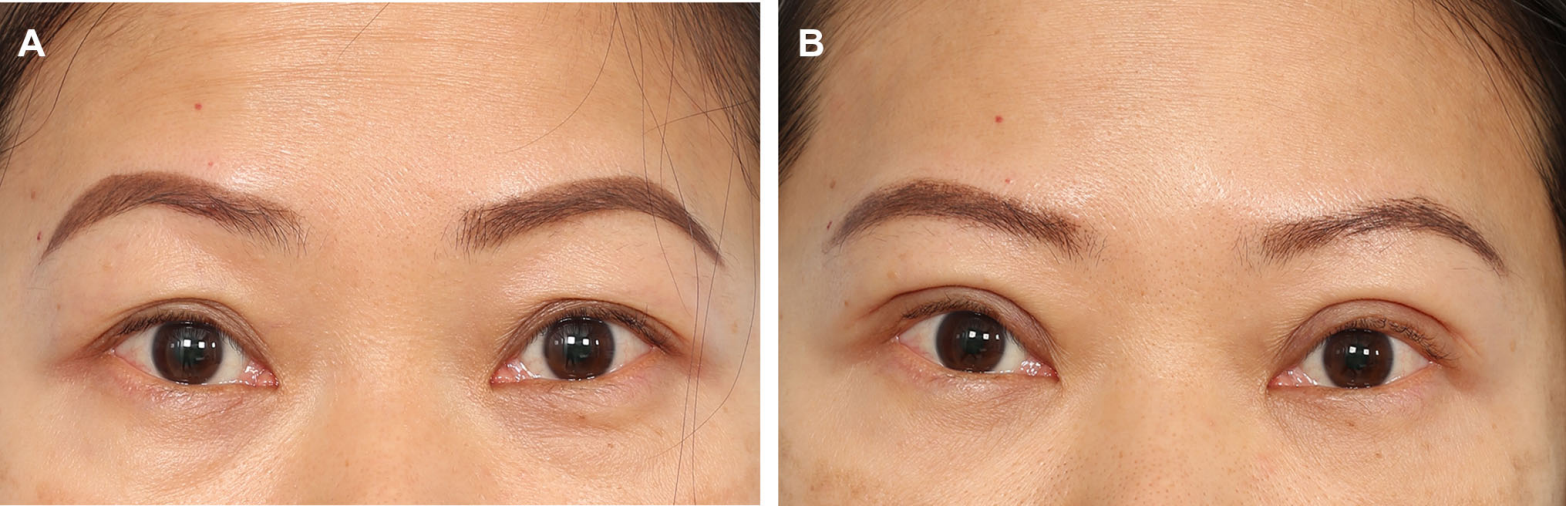
**A** and **B** A 43-year-old female presented for “cosmetic” blepharoplasty. She did not complain of any symptoms of difficulty opening her upper eyelids. On examination, her MRD 1 was normal bilaterally at + 4.5 mm. However, there was clear frontalis straining with eyelid opening. Her right brow elevation was moderate and left was moderate minus. This is a classic case of subclinical upper eyelid ptosis commonly seen in Asian patients. If a blepharoplasty is done without any levator advancement, she will likely develop worsening of the upper eyelid ptosis and upper eyelid asymmetry post-surgery. Bilateral upper eyelid ptosis correction with levator advancement was performed (right – 4 mm, left – 4 mm). Extended transconjunctival eye bag removal was performed at the same time. Here she is shown at 1 year post-surgery. Note that with the surgery, her MRD 1 was maintained at the ideal levels of + 4.5 mm bilaterally while the frontalis straining has been eliminated. Her eyelid creases were crisp and symmetrical with elimination of the multiple crease (right side) and correction of the early A-frame deformity that was present pre-operatively.

**Fig. 7:**
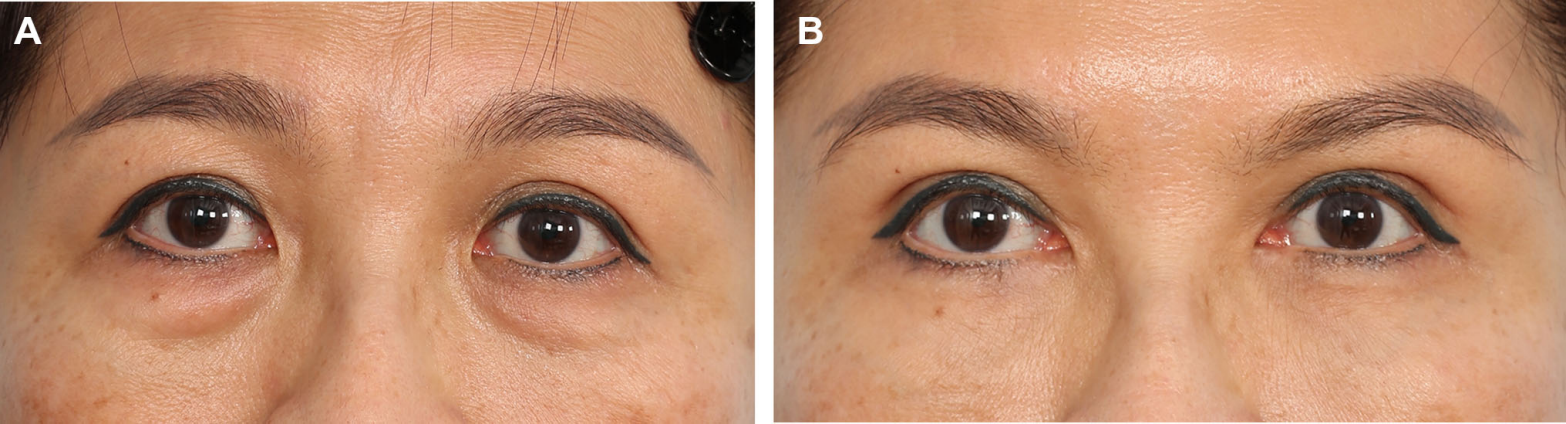
**A** and **B** A 55-year-old female presented for blepharoplasty. On examination, she has straining and difficulty opening her upper eyelids and bilateral frontalis straining with eyelid opening. A levator advancement (right – 3 mm and left − 4 mm) and extended transconjuctival eye bag removal was performed. Here she is shown at 1 year post-surgery. Note the symmetrical and adequate palpebral aperture, symmetrical eyelid crease and complete relaxation of the severe frontalis strain present pre-surgery. These aesthetic and functional effects is possible with proper pre-operative diagnosis of subclinical ptosis and incorporating a precision levator advancement into the upper eyelid procedure

**Fig. 8:**
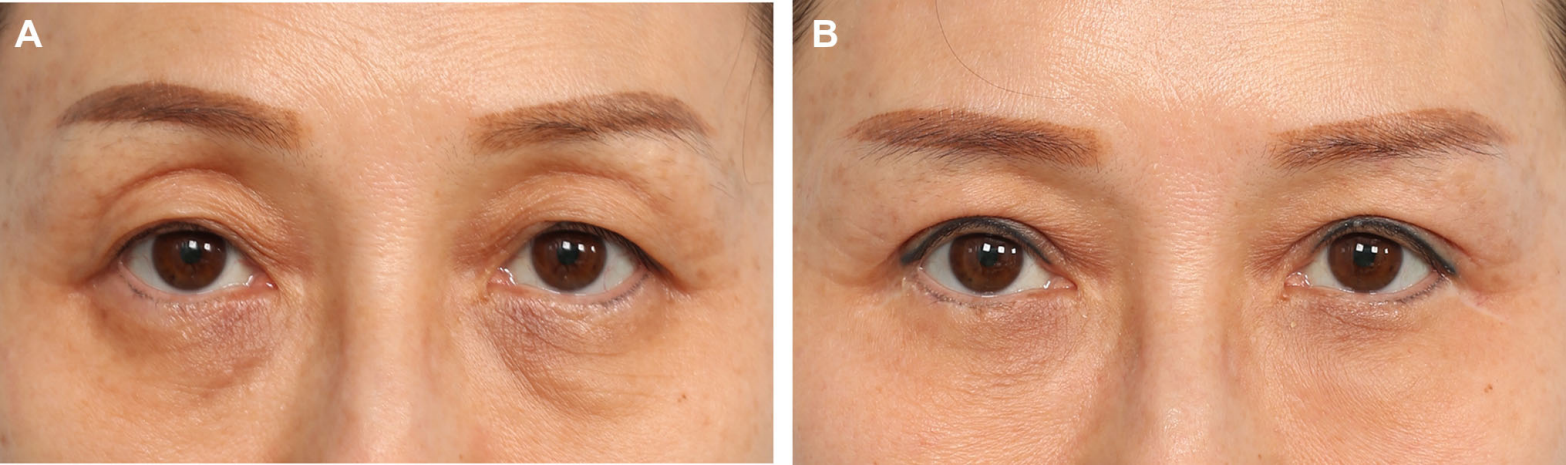
**A** and **B**: A 52-year-old female presented for blepharoplasty. Although her palpebral aperture pre-surgery was quite adequate (at + 4.0 mm on the right and + 4.5 mm on the left), this seemingly “normal” aperture was clearly held at this position by contribution of the frontalis activation. She has significant brow elevation with eye opening and supraorbital hollowing as a result of activation of the frontalis. She has classic subclinical ptosis. Bilateral upper blepharoplasty with levator advancement (right at 0 mm and left – 2 mm) and lower blepharoplasty with mid-cheek lift was performed. Here she is shown at 1 year post-surgery. Note the symmetrical aperture and upper eyelid crease with complete relaxation of the frontalis strain that was very significant pre-surgery. The supraorbital hollowing (A-frame deformities) as a result of the frontalis activation has resolved with the relaxation of the frontalis without the need for upper eyelid fat grafting

**Fig. 9:**
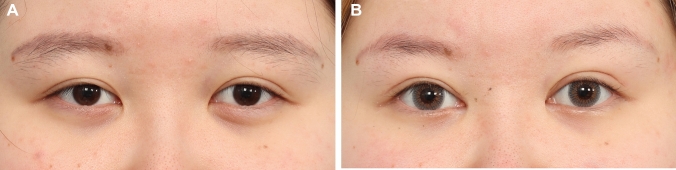
**A** and **B** A 23-year-old female presented with upper eyelid asymmetry and straining to keep her eyelids open. She underwent a cosmetic upper blepharoplasty 4 years ago. On examination, she had slightly narrowed palpebral aperture (MRD 1 of + 4.0 mm and + 3.5 mm on the right and left, respectively) and bilateral frontalis strain with eyelid opening. She noted that she felt that her eye opening was more difficult after the initial upper blepharoplasty. This patient experienced exacerbations of her mild eyelid ptosis after the initial blepharoplasty. This is a classic case of post upper blepharoplasty syndrome (PUBS). A revision upper blepharoplasty with levator advancement was done (right − 2 mm, left − 4 mm). A medial epicanthoplasty was done at the same time. Here she is shown at 18 months post-surgery. Her palpebral apertures have been optimized. The frontalis strain has been eliminated. Subjectively, she reported that the difficulty opening her eyes has resolved after the surgery

Majority of the patients were satisfied with the results with all patients reporting that their appearance has either improved or markedly improved at 1-year follow-up. The formula was able to predict the correct fixation location is 132 of the 192 eyelids analysed (69%). It was able to correctly predict the correct placement location to within +/− 1 mm in 180 of the 192 eyelids analysed (94% of patients). In 98 % of patients, the fixation location was within +/− 2 mm of the estimated location. Figures 2, 3, 4, 5, 6, 7, 8 and 9 demonstrate our results in a range of patients. Figures 2, 3, 4, 5 and 6 demonstrate outcomes in *younger patients* with subclinical eyelid ptosis. The levator advancement delivered superior aesthetic as well as functional outcomes for these patients. Figures 7 and 8 show *older patients* with subclinical ptosis. The need for the levator advancement is easier to understand in these patients. While their pre-operative MRD 1 is normal or “near normal”, usually more severe degree of frontalis strain is evident and it would be quite obvious that their aperture is held in its pre-operative level by the activation of the frontalis muscle. In these patients, the aim of the levator advancement is to adequately restore the intrinsic eye opening, negating the need for the frontalis compensation while precisely controlling the desired palpebral aperture. Post-surgery, the aperture is effectively maintained or only slightly enhanced while the frontalis strain would be significantly eliminated. The final category would be patients that present with subclinical ptosis after previous blepharoplasty (Fig. 9). Cosmetic upper blepharoplasty has the effect of exacerbating the mild ptosis patients may have pre-operatively. This would manifest post-surgery with worsening of the upper eyelid asymmetry and frontalis straining. This is also known as post-upper blepharoplasty syndrome (PUBS) [25]. These patients may be treated effectively with precision levator advancement as presented here. Video #4 shows the recovery and long-term cosmetic and functional results of our surgical demonstration patient. Video #5 demonstrates the long-term cosmetic and functional results of some of our patients (presented in Figs. 4, 6, 7 and 8). Our revision rate was 3% (3 of the 97 patients). All 3 patients required minor revisions of removal of residual skin excesses only. None of our patients required revision of the palpebral aperture.

## Discussion

We have previously published and validated our approach to upper eyelid ptosis correction in Asian patients using the musculoaponeurotic junction formula [17, 18]. The advantage of this approach is that it enables the surgeon to quite accurately determined the location for the levator advancement suture placement pre-operatively from a constant anatomical landmark. Having this information is invaluable as the surgeon would know *exactly where to start* and *exactly how to proceed* to adjust the fixation (if needed) to achieve the desired correction. We have refined our formula to smaller increments of +0.5 mm in the elevation of the eyelid required (Parameter A) and the degree of brow elevation observed with eye opening (Parameter B) [17, 18, 23]. In our experience reported here, this refined formula has greater accuracy in estimating the fixation locations and hence less need for intraoperative adjustments. Increased accuracy is needed to perform the levator advancement in patients with subclinical ptosis. In these patients, the margin for error is narrower, and therefore, greater precision in the pre-operative planning is important for successful outcomes.

Subclinical ptosis is a *compensated state* of eyelid opening in which the upper eyelid is maintained at normal or near normal position by activation of the frontalis muscle [26, 27]. In the literature, “subclinical” or “latent” blepharoptosis is not a clearly defined entity with varied descriptions [2, 15, 28–32]. Choi et al. described subclinical ptosis as when the “upper lid level is more than 1 millimetre below the proper level of the cornea” as suggestive of its presence [29]. Kim et al. stated that objective blockage of the superior cornea of > 2 millimetre by the upper lid as diagnostic of subclinical ptosis. They also alluded that a *pre-operative* frontalis strain as identifiers of subclinical ptosis [28]. Li et al. suggested that the clinical findings of slightly asymmetrically high skin crease, eyebrow position or eyelid margin were diagnostic of “latent aponeurotic ptosis” [30]. These descriptions would be consistent with the definition of subclinical upper eyelid ptosis as used in this paper.

Subclinical ptosis is much more common is Asian patients [2]. The reason for this is anatomical as most Asian patients have a less robust eyelid opening mechanism compared with Caucasian patients. The levator aponeurosis inserts into the antero-superior surface of the tarsus. In patients with crisp, naturally occurring upper eyelid creases, fibres originating from the lower edges of the levator aponeurosis inserts into the dermis at the location of the eyelid crease to invaginate the skin and form the eyelid crease with eyelid opening [14, 33, 34]. *Both these anatomical attachments*, the anterior tarsus and dermis, contribute to the ability of the levator palpebrae superioris to elevate the upper eyelid. Patients with absent or poorly formed eyelid crease have suboptimal eye opening for the following reasons: (1) The absent or poorly developed dermal attachment weaken the ability of the levator palpebrae superioris to open the eyelids and (2) The poorly formed dermal attachments to the upper eyelid skin are surrogate indicators that the quality of levator aponeurosis to the anterior tarsus attachments are less robust. Therefore, these patients may inherently have a weaker eye-opening ability from young. Commonly, these are also patients that present for Asian upper blepharoplasty. Therefore, the need for subclinical ptosis correction among young Asian patients may be *quite high* [28, 30, 32]. Kim et al. reported that 30% of young Korean patients presenting for cosmetic blepharoplasty would require their subclinical ptosis treated [28]. In older patients, a higher percentage would require the levator advancement due to involution weakening from a baseline that was already less than optimal [16]. Therefore, for older Asian patients presenting for either primary or revision upper blepharoplasty who fulfil the diagnostic criteria for subclinical ptosis will require the levator advancement to achieve good aesthetic and functional results.

**Fig. 10:**
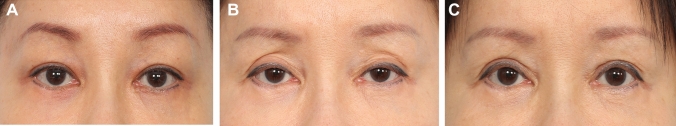
**A** This case illustrates the development of *post-upper blepharoplasty syndrome* (PUBS) and the treatment of this with precision levator advancement. A 55-year-old female patient was seen requesting for upper blepharoplasty. She has a near normal MRD 1 of + 4.0 mm on the right and left, respectively. However, she has significant bilateral brow elevation with eye opening. She has subclinical upper eyelid ptosis and upper blepharoplasty with levator advancement would be the appropriate procedure. **B** Here she was seen again at age 62 (7 years later) having undergone a “cosmetic” upper blepharoplasty by another surgeon 2 years prior. The crease creation/skin excision type cosmetic upper blepharoplasty has resulted in a classic post-upper blepharoplasty syndrome (PUBS). Her lid margins have dropped from further weakening of the levator attachments post-surgery. Her upper eyelid creases were asymmetric and poorly formed due to the levator dehiscence and her brow remained elevated with eye opening from compensation of the frontalis due to the insufficiencies of the intrinsic eyelid opening ability. Hollowing of the upper eyelid/skeletonization was also evident. **C** A revision upper blepharoplasty with levator advancement was performed. In our pre-operative assessment, levator advancements of on the right of − 2.5 mm (− 3.5 + 1.0 + 0) and left of − 0.5 mm (− 3.0 + 1.5 + 1) were planned. Intraoperatively, symmetry and adequate aperture was achieved at − 3.0 mm and − 1.0 mm on the right and left upper eyelid, respectively. Here she is shown at age 63, 1-year post-revision upper blepharoplasty with levator advancement. Note her ptosis has been corrected, the upper eyelid crease is crisp and her brows more relaxed and symmetrical. Her upper eyelid hollowing has also been corrected without the need for fat grafting

Clinically, *most younger patients are asymptomatic*. Older patients and patients for revision blepharoplasty may complain of heaviness, straining to keep their eyes open and even headache towards the end of the day [28, 35, 36]. A key diagnostic feature is the frontalis strain with elevation of the brow with eyelid opening [35]. Incorporating the levator advancement into their upper eyelid surgery is beneficial for two reasons. First, patients with suboptimal eyelid opening at the lower range of normal will benefit aesthetically and functionally with slight tightening of the levator mechanism. This creates wider apertures which are more attractive/brighter with more effective eyelid opening. Secondly, cosmetic upper blepharoplasty, with the associated dissection and post-operative swelling, done in this group of patients quite often will result in attenuation or further weakening of the eyelid opening mechanism. This is manifested by various degrees of asymmetry in the palpebral aperture, worsening of the compensatory elevation of the brow with eye opening resulting in exacerbation of brow asymmetry and poorly formed or uneven upper eyelid creases. This has been called *post-blepharoplasty syndrome* (PUBS) by Steinsapir and Kim [25]. They noted the cause of PUBS to be due to detachment of the levator insertion onto the tarsus from scarring and contracture of the orbital septum post-surgery. Therefore, addressing the subclinical ptosis at the primary surgery is needed to *prevent the development of PUBS*. The revision rates for cosmetic blepharoplasty are reported to be as high as 12–52% [25, 37, 38]. Commonly, the reasons for these revisions include asymmetry of the upper eyelid creases, poorly formed creases or asymmetric apertures. A great majority of these cases may have post-upper blepharoplasty syndrome (PUBS) as an identifiable cause [25]. The treatment for this is to incorporate levator advancement into the revision upper blepharoplasty to optimize both the aesthetic and function aspects of the upper eyelids (Fig. 10).

The advantages of this approach *over conventional upper blepharoplasty* approaches (that focuses on crease creation only in this group of patients) are that the aesthetic results attainable are superior and more predictable. In addition to asymmetries in the upper eyelid creases, minor pre-operative asymmetries in the aperture width, which are quite commonly seen, may be addressed at the same time as well *with the levator advancement*. As importantly, functionally, patients will notice that their upper eyelid opening is more effective and easier after the surgery. Most patients are very appreciative of this functional improvement. The disadvantages are that the surgery is slightly more complicated and the dissection slightly more extensive. Swelling and bruising are slightly more, but with meticulous technique and post-surgical care, this may be minimized. The precision levator advancement required for this technique also has a *steeper learning curve* than conventional approaches.

## Conclusions

The advantages of our approach described in this paper in Asian patients with subclinical ptosis are as follows: (1) The aesthetic results attainable is superior to techniques that focus on crease formation only, without direct manipulation of the levator mechanism. This technique widens *and* equalizes the palpebral apertures. It can deliver a more symmetrical and crisp upper eyelid creases, and eliminate frontalis strain while relaxing the brows to a more aesthetic and symmetrical positions. (2) Functionally, the intrinsic eye-opening mechanism is also optimized. Patients feel less peri-ocular strain and are able to open their eyelids more effectively, eliminating the sensation of straining needed to keep the eyelids open. Patients are profoundly appreciative of these functional benefits. It is important to realize that in the upper eyelid, function *and aesthetics are intricately related* [39]. Thus, it is necessary to optimize function to be able to achieve the desired cosmetic results predictably. This understanding represents a paradigm shift in Asian upper blepharoplasty.

### Supplementary Information

Below is the link to the electronic supplementary material.

Video #1: This video explains our system of assessment of brow elevation with eye opening (MP4 57170 kb)

Video #2: This video shows our pre-operative determination of levator advancement fixation locations relative to the musculoaponeurotic junction (MAJ) based on our system of assessment (Table 1) for our surgical demonstration patient (MP4 41275 kb)

Video #3: This video shows our surgical technique (MP4 124762 kb)

Video #4: This video shows the recovery process and long-term cosmetic and functional results of our surgical demonstration patient (MP4 31351 kb)

Video #5: This video shows the functional outcomes of our patients presented in Figs. 4, 6, 7 and 8 (MP4 39006 kb)
